# Equity-Oriented Healthcare: What It Is and Why We Need It in Oncology

**DOI:** 10.3390/curroncol29010018

**Published:** 2022-01-04

**Authors:** Tara C. Horrill, Annette J. Browne, Kelli I. Stajduhar

**Affiliations:** 1 School of Nursing, University of British Columbia, Vancouver, BC V6T 2B5, Canada; annette.browne@ubc.ca; 2 School of Nursing, University of Victoria, Victoria, BC V8P 5C2, Canada; kis@uvic.ca

**Keywords:** health equity, healthcare accessibility, health services, cultural safety, cancer

## Abstract

Alarming differences exist in cancer outcomes for people most impacted by persistent and widening health and social inequities. People who are socially disadvantaged often have higher cancer-related mortality and are diagnosed with advanced cancers more often than other people. Such outcomes are linked to the compounding effects of stigma, discrimination, and other barriers, which create persistent inequities in access to care at all points in the cancer trajectory, preventing timely diagnosis and treatment, and further widening the health equity gap. In this commentary, we discuss how growing evidence suggests that people who are considered marginalized are not well-served by the cancer care sector and how the design and structure of services can often impose profound barriers to populations considered socially disadvantaged. We highlight equity-oriented healthcare as one strategy that can begin to address inequities in health outcomes and access to care by taking action to transform organizational cultures and approaches to the design and delivery of cancer services.

## 1. Introduction

Despite advances in research and practice, and publicly funded healthcare in Canada, alarming differences exist in cancer outcomes for people most impacted by persistent and widening health and social inequities (herein, people who are marginalized) [1,2]. We use the term ‘marginalized groups’ to refer to groups who experience significant health and social inequities as a result of social, economic, political and historical conditions, and to signify that these inequities disproportionately affect particular segments of the population. This includes, for example, people experiencing the often intersecting impacts of racism and other forms of discrimination, poverty, mental illness, substance use issues and related stigma, homelessness, and disability [2,3]. In addition to extensive co-morbidities, accelerated aging, and premature death [4,5], people who are marginalized experience higher cancer mortality and are diagnosed with advanced cancers more often than other people [6,7,8,9,10,11,12,13]. For example, people with severe mental illness have 92% higher odds of an advanced cancer at diagnosis than those without mental illness, while cancer-related deaths are two times higher among homeless adults and 1.7 times higher among individuals with severe mental illness compared to the general population [6,8]. Such outcomes are linked to the compounding effects of stigma and discrimination, often unintentional, and other barriers, which create persistent inequities in access to care at all points in the cancer trajectory, preventing timely diagnosis and treatment, and further widening the health equity gap [13,14,15,16,17,18,19,20,21,22,23,24].

Increasingly, the cancer care system is known to prioritize a biomedical model of service delivery, concentrating on physiological and protocol-based standardized care, often at the expense of addressing psychosocial and other needs [17,25,26]. In particular, cancer services are sometimes recognized as narrowly focused on active cancer treatment and have been acknowledged as unwelcoming environments for people who are marginalized, with services that are not designed to be flexible or accommodating to the needs, social contexts, and experiences of marginalized people [17,23,25,27,28]. For example, in our experiences as researchers and clinicians, we have seen how people who experience significant inequities are not able to access or proceed with appropriate treatment because they are unable to adhere to highly rigid schedules of chemo- or radiation therapy [17,29]. Moreover, within the current structure of the cancer care system, services are typically not designed to foster a sense of trust or emotional safety, align with the social-emotional needs of patients, or work in ways that explicitly aim to make patients who experience marginalization feel accepted and respected [17,19,25,28].

Indeed, there is growing evidence that people who are socially disadvantaged are not well-served by the cancer care sector [3]. For example, people who experience poverty and unstable housing are significantly less likely to be seen by an oncologist after a cancer diagnosis and are significantly less likely to receive cancer treatment [21]. Similarly, individuals with severe mental illness (e.g., schizophrenia) are two times less likely to receive guideline-recommended cancer treatment [8]. In their work on cancer disparities among individuals with mental illness, Davis et al. note that “differences in cancer care delivery from the point of diagnosis may explain the higher risk of death and may be a modifiable means of improving outcomes” [7] (p. 90). To close this health equity gap, addressing these challenges should be an urgent priority.

## 2. Health Equity and the Cancer Care Sector

Recently, we have noted growing calls to prioritize health equity and redress inequities from within the cancer care sector. Health equity is often defined in relation to health inequities, which are unjust and modifiable differences in health, quality of life, and well-being between and within groups [30]. In 2020, the American Society of Clinical Oncology (ASCO) published a policy statement highlighting health equity as a key concern, focusing on the need to improve equitable access to care and address structural barriers; a strategic plan for achieving health equity across the cancer continuum currently in development [31,32]. In Canada, equity is identified by the Canadian Partnership Against Cancer (CPAC) as one of its five strategic themes for 2017–2022 [33]. In its most recent cancer control strategy, the Partnership highlights “eliminating barriers to people getting the care they need” as one of five priorities for the next decade [3] (p. 26). The strategy also states it will address racism, discrimination, and stigma through healthcare provider education and training.

In Ontario, one of Canada’s largest provinces, the Ontario Cancer Plan [34] identifies equity as one of 6 overarching aims, with a specific goal to “improve health equity across the cancer system such that people are not disadvantaged by who they are, where they live, or what resources they have”. How this goal will be operationalized is not entirely clear. In Manitoba, in recognition of the burden of health inequities impacting the high proportions of Indigenous peoples residing in this province, the Roadmap to Cancer Control calls for acknowledgment that racism and culturally safe care must be addressed in order to provide equitable care [35]. However, none of the stated objectives within the Roadmap address upstream determinants of health, nor do they explicitly address systemic racism or discrimination. The exception is a statement in relation to Indigenous Peoples: “Providing culturally responsive, equitable care requires an understanding of First Nations, Métis and Inuit worldviews and a holistic approach to their health and wellbeing. It also requires efforts to reduce and eliminate the impact of racism within the cancer care system and at points of care” [35] (p. 52).

While we are encouraged by the burgeoning calls to address health equity within the cancer care sector, it seems that the ways in which equity is conceptualized within calls to action, roadmaps, or strategic plans may not sufficiently address the root causes of health inequities. For example, strategies to improve health equity often include increasing investments in healthcare services with the assumption that this will lead to increased *use* of these services, without necessarily attending to *how* these services are designed and delivered [36]. Yet evidence suggests that the design and structure of health services are equally, if not more, important, and “can impose a profound barrier to socially disadvantaged populations who may end up systematically excluded from programs, resulting in worsening of health inequities” [23] (p. 552). Moreover, although some cancer control plans include goals of addressing racism and discrimination, they do not include concrete steps or transformative actions beyond healthcare provider training. While an important component of improving health equity, such training will be ineffective if not complemented by organizational and systems-level shifts in policy and practice [37,38,39].

## 3. Equity-Oriented Healthcare

A key pathway to addressing health equity in the cancer care sector and improving access to cancer care includes integrating approaches to the design and delivery of care that are purposefully responsive to health inequities. While cancer care organizations (and even the cancer care sector as a whole) do not have the power to shape social determinants of health within our societies, they can begin to address inequities in health outcomes and access to care by taking action to transform the organizational culture of healthcare and approaches to service delivery at the point of care [37,40]. The theory and evidence base that underpins equity-oriented healthcare (EOHC) provides one approach to design services that are responsive to inequities [37,41,42,43,44].

EOHC aims to lessen the impacts of structural inequities (e.g., poverty), discrimination, stigma, and racism while reducing mismatches between current approaches to healthcare and the actual needs of people affected by inequities [37,45]. Rather than focusing on a specific ethnocultural group, or being driven by a single demographic variable or segment within the population (e.g., immigrants, South Asian populations, older adults), EOHC refers to an approach that takes into account: the effects of structural inequities, including the inequitable distribution of the determinants of health (such as poverty, lack of affordable housing); the impact of multiple and intersecting forms of racism, discrimination and stigma (e.g., related to mental illness, substance use, non-conforming gender identities, etc.) on people’s access to services and their experiences of care; and the frequent mismatches between dominant approaches to care and the needs of people who are most affected by health and social inequities [37].

In the Canadian-led, interdisciplinary research program known as EQUIP Healthcare (www.equiphealthcare.ca, accessed on 21 November 2021) transformative equity-oriented care is based on 3 key dimensions:

- Trauma- and violence-informed care (TVIC): recognizing and limiting the effects of trauma and violence, including structural violence, on peoples’ lives, health, and care experiences;
- Culturally-safe care/anti-racist approaches: reducing power imbalances, systemic racism, and discrimination;
- Harm reduction/substance use health: focusing on preventing harms from substance use and intersecting forms of stigma [37,43].

Tailorable to diverse settings and contexts, these three key dimensions can be used to guide the development of organizational and point-of-care interventions, strategies, policies, and strategic plans to mitigate the impact of health inequities and support the implementation of equity-oriented approaches [46].

In Canada, EOHC has been taken up in several healthcare sectors including primary care [37,43,47,48], emergency departments [49,50], gender-based violence [46] and in relation to people experiencing substance use stigma [46,51]. There is also movement within Canada to integrate equity-oriented care principles into the design and delivery of palliative care services [52]. Although research is ongoing, in the primary care sector, EOHC approaches have been shown to be predictive of improved self-reported health outcomes and improved care experiences among patients who are marginalized [43].

These impacts, however, are contingent on alignment between the aims of EOHC at the *organizational* level, and the organization’s mandate and strategic long-term goals. Indeed, evidence suggests that although some individual healthcare providers attempt to integrate equity-oriented approaches to care within the cancer care sector, there is a lack of capacity at the organizational level to support the delivery of these types of care [38]. Our ongoing work is aimed at better understanding the organizational contexts and conditions required to optimally support the integration of equity-oriented approaches to the design and delivery of cancer services [53].

Indeed, both ‘bottom-up’ and ‘top-down’ strategies are needed [52]. ‘Bottom-up’ strategies at the level of service delivery include delivering care that is trauma- and violence-informed and culturally safe, with specific attention to how racism, stigma, social dynamics, and unequal relations of power play out, often inadvertently, in patient-provider interactions. Delivering such care will require healthcare providers to be trained in foundational concepts including anti-racist practice, cultural safety, and structural determinants of health, and until recently, these concepts have not been widely included in health professional education [16,54,55]. Although outside of the scope of the cancer care sector, including education on these concepts into health professional training and continuing competency requirements should be a priority [16]. Indeed, although there is increasing awareness of concepts such as cultural safety among healthcare providers in Canada, integration of cultural safety training into health professional training programs has had limited success, and work to address this issue is ongoing [56]. More importantly, educational interventions alone are insufficient to transform healthcare organizations, let alone health systems [39].

‘Top-down’ strategies—those that examine and modify the organizational structures, processes, and policies that shape access to and the delivery of healthcare services—are equally important in addressing health inequities. This may include providing leadership at all levels in support of health equity initiatives, adapting care pathways to more optimally support people who are marginalized, or modifying appointment booking policies or processes for the management of waiting rooms and clinical spaces. Supporting healthcare providers to deliver care that is trauma- and violence-informed and culturally safe, as discussed above, requires organizational commitment, including policy directives, and mechanisms for accountability [39]. These types of strategies fall within the domain of cancer care organizations and would represent a starting place to meaningfully address inequities within cancer care.

For example, within the cancer care sector, patients experiencing significant social disadvantage may encounter the “3 strikes” policy. Although not necessarily a formal policy, oncology care providers describe clinical scenarios in which patients are denied access to diagnostic services (e.g., colonoscopy, biopsy) or not permitted to continue with treatment (e.g., chemotherapy, radiotherapy) if they miss three appointments, with little consideration of the context (e.g., housing instability, extreme poverty) or individual circumstances (e.g., personal history of trauma or abuse). Such policies not only limit patients’ access to care but also limit healthcare providers’ ability to tailor care to individual patients and respond to inequities. Integrating EOHC strategies into the cancer care sector may involve providing wrap-around services to address determinants of health (e.g., providing housing or shelter by partnering with local organizations, providing social work support), and tailoring care to address other specific challenges (e.g., modifying procedures to support patients who are survivors of abuse), while *also* revisiting and revising existing policies through an equity lens. To be clear, we are not suggesting that the cancer care sector ought to address all health and social inequities impacting patients diagnosed with cancer. However, in the process of designing and delivering cancer services and in the provision of direct care, we ought to be taking into account the ways in which patients are impacted by various inequities and social disadvantages (e.g., lack of housing and the need to prioritize other aspects of survival) and their ability to “comply with” or “adhere to” prescribed care (e.g., appointment schedules, treatment regimens). These considerations shed light on how policies, organizational procedures, and processes of care can be unfair and inhumane.

## 4. Conclusions

Against a backdrop of deepening health inequities and calls to close health equity gaps within the cancer care sector, meaningful action is urgently needed. EOHC holds great promise as one strategy to address inequities within the cancer care sector. Our aim in this commentary is to prompt organizations, clinicians, decision-makers, and researchers to consider how the organizations and services within the cancer care sector could be transformed in ways that would better align with the needs of people who are socially disadvantaged, particularly those who often fall through the cracks or whose social, economic, and personal circumstances are such that they do not access care that would otherwise be warranted. Although we draw on experiences and examples from within the Canadian context, inequities in cancer outcomes and access to cancer care exist worldwide and require urgent attention [57]. Integrating equity-oriented approaches into the design and delivery of cancer services will go a long way in helping people who experience marginalization feel safe, comfortable, and respected, all of which are key to improving access to cancer services and addressing inequities.

## Data Availability

Not applicable.
